# Early-Life Origins of Metabolic Syndrome: Mechanisms and Preventive Aspects

**DOI:** 10.3390/ijms222111872

**Published:** 2021-11-02

**Authors:** Chien-Ning Hsu, Chih-Yao Hou, Wei-Hsuan Hsu, You-Lin Tain

**Affiliations:** 1 Department of Pharmacy, Kaohsiung Chang Gung Memorial Hospital, Kaohsiung 833, Taiwan; cnhsu@cgmh.org.tw; 2 School of Pharmacy, Kaohsiung Medical University, Kaohsiung 807, Taiwan; 3 Department of Seafood Science, National Kaohsiung University of Science and Technology, Kaohsiung 811, Taiwan; chihyaohou@webmail.nkmu.edu.tw; 4 Department of Food Safety/Hygiene and Risk Management, College of Medicine, National Chen Kung University, Tainan 701, Taiwan; whhsu@mail.ncku.edu.tw; 5 Department of Pediatrics, Kaohsiung Chang Gung Memorial Hospital and Chang Gung University College of Medicine, Kaohsiung 833, Taiwan; 6 Institute for Translational Research in Biomedicine, Kaohsiung Chang Gung Memorial Hospital and Chang Gung University College of Medicine, Kaohsiung 833, Taiwan

**Keywords:** cardiovascular disease, obesity, hypertension, oxidative stress, metabolic syndrome, dyslipidemia, insulin resistance, diabetes, nutrient sensing, developmental origins of health and disease (DOHaD)

## Abstract

One of the leading global public-health burdens is metabolic syndrome (MetS), despite the many advances in pharmacotherapies. MetS, now known as “developmental origins of health and disease” (DOHaD), can have its origins in early life. Offspring MetS can be programmed by various adverse early-life conditions, such as nutrition imbalance, maternal conditions or diseases, maternal chemical exposure, and medication use. Conversely, early interventions have shown potential to revoke programming processes to prevent MetS of developmental origins, namely reprogramming. In this review, we summarize what is currently known about adverse environmental insults implicated in MetS of developmental origins, including the fundamental underlying mechanisms. We also describe animal models that have been developed to study the developmental programming of MetS. This review extends previous research reviews by addressing implementation of reprogramming strategies to prevent the programming of MetS. These mechanism-targeted strategies include antioxidants, melatonin, resveratrol, probiotics/prebiotics, and amino acids. Much work remains to be accomplished to determine the insults that could induce MetS, to identify the mechanisms behind MetS programming, and to develop potential reprogramming strategies for clinical translation.

## 1. Introduction

Metabolic syndrome (MetS) is not a single disease but a collection of medical conditions that occur together and increase the risk of cardiovascular disease (CVD). Although MetS had been defined slightly differently by various organizations [1], the main components of MetS include obesity, hypertension, dyslipidemia and insulin resistance. The prevalence estimates vary, based on the criteria used for the diagnosis of MetS. Thus far, global MetS prevalence is estimated to affect approximately one quarter of the world population [2]. More importantly, MetS-related disorders account for two thirds of the non-communicable disease (NCDs) deaths [3]. Also important is that it displays multiple and diverse phenotypes with indications for differing treatment strategies. In the absence of specific therapeutic regimens, the prevalence of MetS remains on the increase globally [2]. Therefore, a superior strategy to stop the spread of the MetS epidemic is required to for prevention, in addition to treatment.

Most NCDs may start in early life [4]. The establishment of various adverse environmental conditions, which takes place during pregnancy and lactation, could be involved in the early-life programming of health. This concept is now known as the developmental origin of health and disease (DOHaD) [5], which is supported by evidence coming from a large number of epidemiological and experimental observations.

Various early-life risk factors often linked to nutritional imbalance may lead to vulnerability to later MetS [6,7,8,9,10]. Remarkably, there is evidence for the programming of some similar features of MetS from different early-life insults, possibly suggesting a commonality of mechanism and highlighting that identifying underlying mechanistic pathways is vital to develop ideal prevention interventions [6]. By intervening before disease ever occurs, we have the potential to stop undesirable programming processes resulting in MetS, which is referred to as reprogramming [11].

Our review aims to map the key concepts in the developmental programming of MetS. Hence, we first highlight epidemiological studies that link early-life factors to MetS of developmental origins. This is followed by a summary of the potential developmental mechanisms behind the origin of MetS. Furthermore, we present a summary of reprogramming interventions from animal studies that can prevent the development of MetS.

Our search strategy was designed to retrieve published manuscripts in the English language from January 1980 to September 2021 relating to MetS and DOHaD from the PubMed/MEDLINE databases. We used different combinations of the following search terms: “cardiovascular disease”, “cardiometabolic disorder”, “developmental programming”, “DOHaD”, “reprogramming”, “dyslipidemia”, “hyperlipidemia”, “obesity”, “diabetes”, “insulin resistance”, “hyperglycemia”, “hypertension”, “mother”, “father”, “paternal”, “pregnancy”, “gestation”, “offspring”, “progeny”, and “metabolic syndrome”. Additional papers were then selected and evaluated on the basis of fitting references in eligible literature.

## 2. Epidemiological Evidence Linked Early-Life Insults with Offspring MetS

Extensive epidemiological studies have linked adverse early-life conditions with the risk of MetS in the offspring in later life. First, there is evidence from severe famines [12,13,14,15,16]. The Dutch Famine Birth Cohort Study demonstrated that the children of pregnant women exposed to famine displayed various characteristics of MetS, like obesity, dyslipidemia, hypertension, insulin resistance and CVDs [12,13]. Findings similar to those of the Dutch famine were also established in other famine studies [14,15,16]. Of particular interest, maternal undernutrition during early gestation had greater effects in increasing cardiometabolic risk in adult offspring, compared to maternal undernutrition during middle- or late-gestation [12]. Another line of evidence comes from research in twin pregnancy. These studies reported there were associations between low birth weight (LBW) and different characteristics of MetS, including hypertension, type 2 diabetes and insulin resistance [17,18]. Third, a prior systematic review of 39 studies revealed that rapid postnatal catch-up growth of LBW neonate had an approximately 80% increased risk for CVDs, a major complication of MetS [19]. The risk factors for MetS of developmental origins have been assessed in a number of observational studies. Risk factors reported in these cohorts relating to adverse cardiometabolic outcomes in adult offspring include maternal obesity [20,21], gestational diabetes [21,22], excessive postnatal weight gain, in [23] and environmental chemicals exposure [16]. To date, literature on paternal risk factors is scare. There is emerging evidence that paternal risk factors, such as obesity, diabetes mellitus, advanced age, and cigarette smoke are associated with adverse metabolic and cardiovascular outcomes in their offspring [24,25]. All of these observations provide links between the suboptimal early-life environment and the risks for developing MetS in adulthood.

However, these epidemiological studies do not propose molecular mechanisms underlying programming processes for the development of potential reprogramming interventions. Therefore, animal models have been established to demonstrate the biological plausibility of the associations observed in epidemiological studies, providing proof of causality.

## 3. Animal Models for Developmental Origins of MetS

In view of the difficulties in establishing animal models that manifest all the characteristics of MetS, most investigations into MetS of developmental origin are done using models that display certain hallmarks of MetS [6,7,8]. To date, a broad range of adverse early-life environmental factors are associated with certain features of MetS in adult offspring, including nutrition imbalance, maternal conditions or disease, chemical exposure, medication use, etc. [6,7,8,9,10,26,27,28,29]. However, excessive information is available based on only one characteristic of MetS, and in the interest of brevity, we have limited this review to at least two of the listed components that are present [30,31,32,33,34,35,36,37,38,39,40,41,42,43,44,45,46,47,48,49,50,51,52,53,54,55,56,57,58,59,60,61,62,63,64,65,66,67,68,69,70,71,72] (See Table 1). Diverse small- and large-animal models have been developed for DOHaD research [29,73], each with its own natural advantages and disadvantages. This review was restricted to rodent models for appropriate comparisons of major characteristics of MetS appear through a lifetime. Table 1 reveals the outcomes relating to MetS determined in rats ranging from eight weeks to one year of age. Considering one human year is almost equivalent to two rat weeks in adulthood [74], most outcomes are determined from childhood to middle adulthood in terms of human age. Each category is discussed in turn.

### 3.1. Nutritional Imbalance

Studies of nutritional programming linking DOHaD-related disorders using animal models have been ongoing since the early 1990s [73]. Dietary manipulation has been the focus of a large body of work relating to MetS of developmental origins. Table 1 shows insufficient or excessive consumption of a certain nutrient has been used to induce different features of MetS. Following the studies of the Dutch famine [12,13], a number of maternal nutrient restriction models have been established to mimic the undernutrition experienced by pregnant women at that time.

Caloric restriction is defined as an overall reduction in energy and nutrient intake without incurring specific nutrient. In rats, a 50% caloric restriction during gestation and lactation can result in hypertension and insulin resistance in adult offspring [28,29,30]. Restriction of calories by a range of 30% to 70% in pregnant rats has been reported to induce increases of BP in their adult progeny, as reviewed elsewhere [74]. From a general perspective, offspring exposed to a greater degree of caloric restriction are prone to develop hypertension earlier [75]. However, the extent to which the severity of caloric restriction affects other characteristics of MetS in adult offspring remains to be elucidated further.

Similar to caloric restriction, the protein restriction model may mimic the challenge faced in developing nations. Adult rat offspring born to dams exposed to protein restriction during pregnancy develop hypertension [31] and insulin resistance [32]. Although a more severe protein restriction causes an earlier development of hypertension in offspring [75], a previous study reported that two low-protein diet manipulations with the same protein concentration (9%) but different components in pregnant rat provoked different programming effects on BP in adult offspring [76]. It is therefore possible that the balance of specific amino acids and other nutrients may be a critical determinant in programming of MetS-related phenotypes but not protein restriction per se.

Table 1 indicates maternal high-fat diet programs almost all feature of MetS in adult rat offspring at 14–16 weeks of age, such as hypertension [33], obesity [34,36], dyslipidemia [35,36], and insulin resistance [36]. A high-fat diet has been widely used to explore the mechanisms of metabolic disease of both established and developmental origins [77,78]. Nevertheless, the programming effects of maternal high-fat diet on offspring BP are diverse according to age, sex, strains, and different fatty acids compositions [78]. Notably, abnormal regulation of insulin signaling and lipid metabolism programmed by a maternal high-fat diet can be promoted by a post-weaning high-fat diet, leading to development of MetS-related phenotypes in adult offspring [37,79,80]. A maternal high-fructose diet has also been reported as a commonly used animal model for studying MetS of developmental origins [81]. We and others have shown that adult rat offspring of mothers exposed to 60% high-fructose diet during gestation and lactation displayed MetS-related comorbidities [39,40,41,81]. Furthermore, mother rats receiving a high-fructose diet plus a high-fat diet saw an elevation in BP and BW in their offspring [42,43]. These findings suggest that maternal diets containing key components based on the human Western diet may have synergistic effects between fat and sugar on the development of features of MetS in adult offspring. Moreover, deficiencies in micronutrients, including calcium [44,45], zinc [46,47], and vitamin D [48,49] in pregnant mother rats are associated with offspring MetS.

### 3.2. Maternal Illnesses and Conditions

Complications during pregnancy and maternal diseases can affect fetal programming, resulting in intrauterine growth retardation (IUGR) in offspring [82]. As IUGR reflects an abnormal adaptive fetal growth in an adverse intrauterine environment, IUGR animal models are applied to decipher the underlying mechanisms behind MetS of developmental origins [82]. In the model of uteroplacental insufficiency developed by uterine artery ligation in the pregnant rat, IUGR offspring developed hypertension, dyslipidemia and insulin resistance in adulthood [50,51]. Additionally, several animal models resembling maternal conditions and diseases have been evaluated, such as polycystic ovary syndrome (PCOS) [52,53], maternal hypoxia [52,54], maternal inflammation [55,56], diabetes [57,58,59], and chronodisruption [60,61].

Epidemiological observations have established that inflammatory disorders, PCOS, and hypoxia increase the risk of pregnancy complications [83,84]. In the PCOS model, maternal hyperandrogenemia was induced by injection of testosterone cypionate in late gestation for studying cardiometabolic outcomes in adult offspring. Accordingly, adult offspring exhibited hypertension and dyslipidemia at 16–17 weeks of age [52,53]. Likewise, several features of MetS can be programmed by maternal hypoxia and inflammation, including hypertension [52,55], obesity [54], and insulin resistance [54,56].

It is clear from a range of human observational studies that maternal diabetes gives rise to different phenotypes of MetS in offspring, including obesity, insulin resistance, hypertension, dyslipidemia, and CVDs [85]. The majority of rodent studies of maternal diabetes have employed streptozotocin (STZ)-induced diabetes [57,58,59]. When injected into neonates [57,58] or adult rats [57,59], STZ can cause type 1 or type 2 diabetes, respectively. Almost all major characteristics of MetS are present in adult offspring born to diabetic mothers at 12–16 weeks of age [57,58,59], which are largely in line with the findings in humans.

There is now increasing evidence connecting disturbances in the circadian rhythm with the key components of MetS [86]. A meta-analysis including 22 studies showed that overall sleep quality as well as sleep complaints have significant positive associations with MetS [87]. The circadian system is the major regulator of human metabolism [88]. The circadian clock system consists of central and peripheral clocks, which are coordinated to produce daily rhythms [89]. This central clock located in the suprachiasmatic nucleus regulates the body’s metabolism through synchronizing peripheral clocks in our body’s key organs such as the heart, kidney, liver, muscle and adipose tissue [90]. Accordingly, it is not surprising that circadian rhythm sleep disorders have been linked to several components of the MetS [86].

In pregnant women, circadian disruption can lead to a wide range of adverse consequences for their children [91]. Maternal circadian disruption affects not only central and peripheral circadian clocks but also a range of endogenous circadian signals including melatonin and glucocorticoid secretion [92,93]. Although data on maternal sleep disorder programs MetS in offspring remain limited, two animal studies have reported that adult rat offspring born to dams received constant light exposure or pinealectomy developed hypertension [60] and insulin resistance [61].

### 3.3. Chemical and Medication Exposures

Relatively few studies have investigated early-life chemical and medication exposures on developmental programming of MetS. A broad range of early-life environmental chemical exposures have been related to increased risk for developing hypertension of developmental origins, as we reviewed elsewhere [94]. These chemicals, di-(2-ethylhexyl) phthalate (DEHP) and bisphenol A, have been studied for their impact on insulin resistance in adult progeny [63,65]. These findings are in agreement with epidemiological research data showing that endocrine-disrupting chemical exposure is linked to CVDs later in life [95].

As with chemical effects during development, substance abuse is another risk factor. A high proportion (6–16%) of pregnant women in the United States are cigarette smokers, alcohol abusers, or illicit drug users [96]. In rodent models, maternal nicotine or alcohol exposure causes hypertension [66,68], insulin resistance [67,69], and obesity [69] in adult offspring.

Despite a number of medications (such as cyclosporine [97] and minocycline [98]) administered in pregnancy that have been related to developmentally programmed hypertension in adult offspring [29], only glucocorticoid has been studied for other features of MetS [70,71,72]. During development, the fetus is at risk of glucocorticoid exposure through excess maternal corticosteroids (e.g., stressed pregnancies) or through exogenous administration (e.g., preterm birth). Antenatal or neonatal administration of dexamethasone lead to hypertension [70,71] and insulin resistance [72] in adult rat offspring.

Comparable to maternal programming, reported animal models relating to paternal factors-induced MetS in offspring are rather limited [25]. So far, only adult offspring of paternal low protein intake [99], paternal high-fat diet [100], and paternal hyperglycemia [101] have been evaluated, and these have developed at least two components of MetS.

Considering animal models are in good agreement with the epidemiological observations showing various maternal insults induce similar features of MetS in offspring, it is possible that various insults may mediate common mechanisms culminating in MetS of developmental origins.

## 4. Common Mechanisms behind Metabolic Syndrome of Developmental Origins

**Figure 1:**
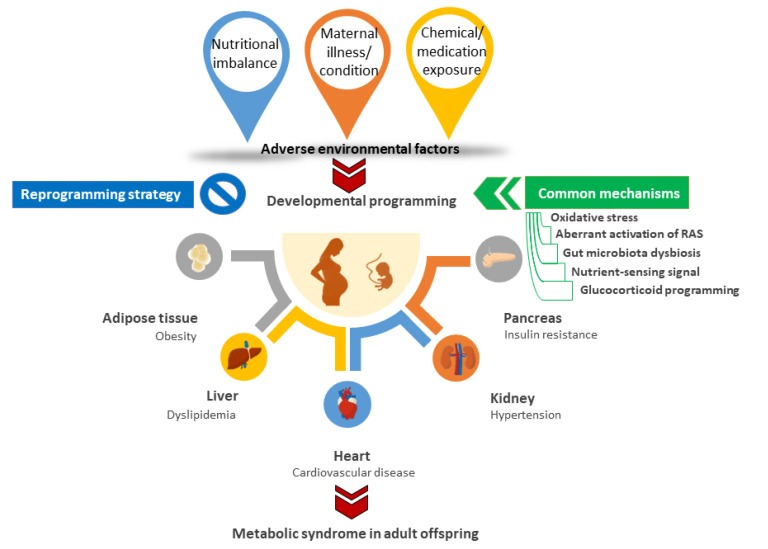
A schematic representation outlining the adverse environmental factors and potential mechanisms that may underlie the developmental programming in different organ systems resulting in metabolic syndrome in adulthood. By switching therapy from adulthood to early life before metabolic syndrome occurs by so-called reprogramming, we have the potential to prevent metabolic syndrome of developmental origins. RAS = renin-angiotensin system.

Though the common mechanisms behind developmental programming of MetS have not yet been thoroughly identified, animal studies have shed light on potential mechanisms, such as oxidative stress [26,102], aberrant activation of the renin–angiotensin system (RAS) [103,104], gut microbiota dysbiosis [105,106], dysregulated nutrient-sensing signals [26], and glucocorticoid programming [107]. Figure 1 is a graphic depiction of early-life adverse environmental factors that mediate common mechanisms and affect multiple interacting organ systems, resulting in MetS of developmental origins in later life.

### 4.1. Oxidative Stress

During development, the fetus is extremely vulnerable to injury to oxidant molecules because of its low antioxidant defense mechanisms [108]. Oxidative stress describes a condition of imbalance in the production of reactive oxygen species (ROS) and the antioxidant defense system, which plays a crucial, mechanistic role in MetS of developmental origins [6,9,109]. As reviewed elsewhere [26], a great number of early-life adverse environmental factors can promote oxidative stress resulting in developmental programming, such as: maternal undernutrition, maternal high-fat diet, maternal high-fructose diet, maternal diabetes, preeclampsia, prenatal hypoxia, maternal inflammation, prenatal glucocorticoid exposure, etc. Complete MetS features can be programmed by maternal high-fructose intake in rodent animal models, as reviewed elsewhere [81]. Among them, hypertension [110], insulin resistance [111], and dyslipidemia [112] have been related to oxidative stress. Current evidence indicates that BP regulation, insulin function axis and lipid metabolic pathways could be sensitive targets to oxidative stress programming [26,102].

Additionally, high levels of ROS can reduce nitric oxide (NO) bioavailability. NO deficiency is a key pathogenic mechanism of MetS-related disorders, such as hyperlipidemia, obesity, diabetes mellitus, hypertension, and CVD [113]. NO deficiency can be caused by inhibition by asymmetric dimethylarginine (ADMA, an NOS inhibitor). Emerging evidence supports that the ADMA-NO imbalance plays a crucial role in the pathogenesis of compromised pregnancies and fetal programming [114]. Among the reasons for MetS, oxidative stress has centered attention on ADMA [114]. Prior research has shown increased plasma ADMA levels are related to major characteristics of the MetS, such as dyslipidemia, diabetes mellitus, hypertension and obesity [115].

Conversely, several agents with antioxidant properties have shown the potential to prevent MetS of developmental origins. Resveratrol is a polyphenolic compound with antioxidant activity [116]. Resveratrol supplementation during gestation and lactation can benefit low protein diet-induced oxidative stress in adult offspring, combined with alleviating characteristics of MetS [117]. Melatonin is also an antioxidant [118]. Its use in pregnancy and lactation has shown beneficial effects on various adult chronic diseases in later life [118]. Previous studies showed maternal melatonin treatment prevents adult offspring against hypertension, glucose intolerance and insulin resistance [60,61]. In view of the fact that MetS is a multi-organ disease, there will be a growing need to better understand organ-specific redox-sensitive signaling responsible for programming processes for MetS of developmental origins.

### 4.2. Aberrant Activation of RAS

The RAS is both a provider and target to MetS [119]. The classic RAS can be defined by the activity of angiotensin-converting enzyme (ACE) to form angiotensin II (ANG II) and the subsequent activation of the Ang II type 1 receptor (AT1R) to promote vasoconstriction. Activation of the classic RAS in specific cell types that mediate various phenotypes of MetS, including hypertension, hyperglycemia and insulin resistance [104,119].

The most common studied phenotype of MetS related to the RAS is hypertension. A variety of animal models listed in Table 1 have indicated the role of RAS, implicating it in renal programming and hypertension of developmental origins. These models include protein restriction [120], high-fat diet [121], high-fructose diet [122], uteroplacental insufficiency [123], maternal hypoxia [124], maternal inflammation [125], diabetes [126], chronodisruption [60], and glucocorticoid exposure [70]. These findings support the importance of the RAS during critical developmental stage on determining hypertension of developmental origins in later life.

On the contrary, early inhibition of classic RAS shows benefits against hypertension of developmental origins [104]. Additionally, RAS inhibition has been reported to control hyperglycemia, insulin resistance and dyslipidemia in patients with MetS [119]. On the other hand, the alternative ACE2/angiotensin-(1-7)/mitochondrial assembly receptor/Mas axis has been identified as a negative regulator of Ang II activity [127]. Considering activation of ACE2/ANG-(1-7)/Mas axis having therapeutic potential in hypertension of developmental origins [104,128], further studies are required to elucidate its reprogramming effects in other MetS-related phenotypes.

### 4.3. Gut Microbiota Dysbiosis

Development of gut microbiota early in life can impact adult diseases of developmental origins [129]. Imbalance of gut microbiota were related to almost all MetS phenotypes, such as hypertension [81,121], obesity [130], insulin resistance [131], and dyslipidemia [132]. Decreased gut microbial richness and diversity are associated with increased risk of developing CVD [133]. Several adverse environmental factors related to MetS of developmental origins have been reported to cause cardiovascular programming coinciding with abnormalities of gut microbiota, including maternal high-fructose diet [134], maternal high-fat diet [121], and maternal PCOS [52].

During recent years, increasing evidence has been accumulated that gut microbiota dysbiosis causes CVD of developmental origins attributed to increases of trimethylamine-N-oxide (TMAO), increases of tryptophan-derived uremic toxins, decreases of short chain fatty acids (SCFAs), and activation of the aryl hydrocarbon receptor (AhR) pathway [106,135,136,137]. SCFAs can modulate glucose homeostasis, appetite regulation and obesity [138]. Many studies report a positive association between increased TMAO level and Met-related disorders [139]. Additionally, several indole derivatives derived from tryptophan by the microbiota may have a role in MetS pathogenesis via activating AhR signaling [140]. These observations support the notion that gut microbiota metabolites are involved in the pathogenesis of MetS of developmental origins.

Conversely, early intervention targeting gut microbiota can prevent CVD in later life [106]. To date, reprogramming interventions based on gut microbiota-targeted modalities include probiotics, prebiotics, and postbiotics [106]. Supplementation with the probiotic *Lactobacillus casei* or prebiotic inulin within pregnancy and lactation protected adult male rat progeny against hypertension programmed by a maternal high-fructose diet [39] or high-fat diet [121]. Another study indicated that maternal oligofructose therapy attenuated hepatic steatosis and insulin resistance induced in adult offspring born to dams received high-fat/high-sucrose diets [141]. We previously demonstrated that maternal 3,3-dimethyl-1-butanol (an inhibitor of TMAO formation) treatment protects adult offspring against maternal high-fructose diet-induced hypertension, which was coincided with the reduction of TMAO levels [134]. Another report showed that maternal acetate supplementation was able to prevent the elevation of BP in adult rat offspring in a high-fructose diet model [36]. Although results from animal studies on microbiota-targeted reprogramming interventions in the prevention of MetS-related disorders are beneficial, we still do not know if these reprogramming effects are organ-specific, and to what extent if they are

### 4.4. Dysregulated Nitrient-Sensing Signal

Nutrient-sensing signals regulate whole-body metabolic homeostasis [142]. During fetal development, nutrient-sensing signals orchestrate fetal metabolism in response to maternal nutritional insults. Accordingly, disturbed nutrient-sensing signals in pregnancy have a unique role in the pathogenesis of MetS of developmental origins [143]. These signals include cyclic adenosine monophosphate (AMP)-activated protein kinase (AMPK), silent information regulator transcript (SIRT), peroxisome proliferator-activated receptors (PPARs), PPARγ coactivator-1α (PGC-1α), and mammalian target of rapamycin (mTOR) pathways [144]. AMPK and SIRT1 can mediate phosphorylation and deacetylation of PGC-1α, respectively [145], to control the expression of PPARs and their target genes. It is known that PPARs govern the expression of specific sets of target genes involved in hypertension of developmental origins [146], which can be driven by maternal nutritional insults.

Conversely, resveratrol, an AMPK activator, can mediate nutrient-sensing signals to activate expression of PPARs target genes and thereby reprogramming MetS-related programmed processes [147,148]. Additionally, AMPK activation by resveratrol treatment protects against insulin resistance and hyperlipidemia in a prenatal hypoxia/postnatal high-fat diet rat model [149]. Furthermore, our prior studies demonstrated that AMPK activation prevents the elevation of offspring’s BP via regulation of nutrient-sensing signals in models of developmental hypertension programmed by a high-fructose diet [150] and a high-fat diet [151]. These observations establish a close connection between nutrient-sensing signals and MetS of developmental origins.

### 4.5. Glucocorticoid Programming

Excess glucocorticoid signaling during pregnancy can disrupt the developmental trajectory of the fetus, resulting in long-term negative consequences [152]. Normally, fetal glucocorticoid levels are much lower than maternal levels due to the protection of placenta barrier via inactivation of glucocorticoid by the enzyme 11b-hydroxysteroid dehydrogenase type 2 (11b-HSD2) [153].

A number of suboptimal intrauterine conditions have linked to inhibit 11b-HSD2, leading to excess glucocorticoid in the fetal stage [152]. Furthermore, exogenous perinatal administration of dexamethasone has been linked to offspring hypertension [70,71] and insulin resistance [72].

Using a prenatal dexamethasone rat model, we analyzed the renal transcriptome in the rat offspring using the whole genome RNA next generation sequencing in which hypertension of developmental origins was studied in adult male offspring [154]. A total of 431 renal transcripts were programmed by dexamethasone exposure at one and sixteen weeks of age. These findings suggest that glucocorticoid programming epigenetically regulated a great number of renal transcripts to contribute to hypertension of developmental origins. Despite detailed mechanisms that behind the epigenetic regulation of these genes and their roles in the programming processes toward development of hypertension remain unraveled, it is conceivable that glucocorticoid programming involves permanent and organ-specific changes in the expression of key genes, resulting in various features of MetS in adult life.

Notably, the placenta is closely interconnected to the aforementioned mechanisms, leading to fetal programming [155]. It is well known that the placenta transports maternal nutrients and oxygen to the fetus for growth and development. Amino acid concentrations in the fetal circulation are tightly controlled by the placenta, which is vital for normal fetal development and health in later life [156]. Placental oxidative stress can cause fetal programming, resulting in adult disease [157]. The placenta not only modulates nutrient-sensing signals [143], but also 11b-HSD2 [153], thereby affecting fetal programming and the long-term health of the offspring. Although the existence and role of the placental microbiome remain inconclusive [158,159,160], it provides a possibility with regard to the alteration of placental microbiome as a possible reprogramming approach in MetS of developmental origins. Additional research on maternal risk factors together with placental programming’s effect on Mets of developmental origins is urgently needed.

## 5. Reprogramming Strategy

With a greater understanding on the mechanisms underlying MetS programming, the development of mechanism-targeted strategies holds potential for reprogramming. So far, strategies to offset underlying mechanisms governing MetS that have been evaluated range from avoidance of risk factors, nutritional interventions, pharmacological therapies, exercise, to lifestyle modification [6,7,8,9,81,161,162].

**Figure 2:**
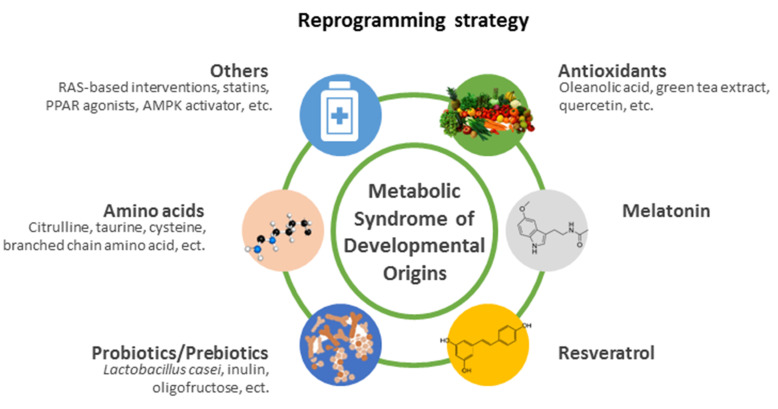
A summary of the currently available reprogramming interventions to prevent metabolic syndrome of developmental origins. RAS = renin-angiotensin system. PPAR = peroxisome proliferator-activated receptor. AMPK = cyclic adenosine monophosphate-activated protein kinase.

This review predominantly focuses on the pharmacological and nutritional interventions as reprogramming strategies to prevent MetS of developmental origins. Given that the difficulties in developing animal models displaying all characteristics of MetS, almost all pharmacological interventions into MetS of developmental origins have been investigated for their beneficial effects against some but not all components of MetS. A schematic summarizing the potential reprogramming interventions for MetS of developmental origins is presented in Figure 2. Each intervention will be discussed in turn.

### 5.1. Antioxidants

Antioxidants can be classified as either enzymatic or non-enzymatic based on their activity [163]. Non-enzymatic antioxidants can be grouped into two categories: natural and synthetic [164]. Examples of natural nonenzymatic antioxidants are vitamin A, C, E, and flavonoids [163]. Most natural antioxidants come from plants. Polyphenols and carotenoids are the two main kinds of antioxidant phytochemicals.

Several phytochemicals with antioxidants have been reported to have beneficial effects against major hallmarks of MetS in adult offspring [161]. A previous report showed that early postnatal administration of oleanolic acid prevented dyslipidemia and insulin resistance in adult rat offspring [165,166]. Likewise, maternal green tea extract supplementation protected adult offspring against dyslipidemia and insulin resistance programmed by a high-fat diet [167]. Another study revealed that high-fat diets induced MetS phenotypes in adult offspring, including obesity, insulin resistance, and dyslipidemia, all of which can be protected by quercetin treatment during gestation and lactation [38]. Moreover, quercetin treatment during pregnancy and lactation reduced adipose tissue mass, improved insulin resistance, and restored dyslipidemia programmed by maternal high-fat diet [38,168].

Although many vitamins and nature-derived antioxidants exert beneficial effects on oxidative stress-related disorders, there is little research about their reprogramming effects on MetS of developmental origins. Considering oxidative stress is a common mechanism behind MetS of developmental origins, there will be a growing need to better understand of the organ-specific mechanisms of various antioxidants in the prevention of various characteristics of MetS.

### 5.2. Melatonin

Melatonin, or N-acetyl-5-methoxytryptamine, is a pleiotropic hormone essential for pregnancy and fetal development [169]. Melatonin is an endogenous indoleamine mainly secreted by the pineal gland at night.

Melatonin is considered as a potential reprogramming intervention, and its benefits on hypertension have been supported by different models such as caloric restriction [170], NO deficiency [171], high-fructose diet [172], chronodisruption [60], and the glucocorticoid exposure model [71]. Additionally, maternal melatonin therapy protected adult rat offspring against obesity and dyslipidemia induced by prenatal dexamethasone administration and postnatal high-fat diet [173]. Notably, prior studies have demonstrated interplay between melatonin and the above-mentioned common mechanisms behind MetS of developmental origins, such as oxidative stress, aberrant RAS, nutrient-sensing signaling, and glucocorticoid programming [118]. These observations support the idea that melatonin may work in different ways to prevent MetS programming [174].

Melatonin is a relatively safe supplement in humans [175]. Although the uses of melatonin during gestation and lactation remain inconclusive, it has been clinically used for certain neonatal diseases [176]. Therefore, further translational research into the long-term MetS-related outcomes of perinatal melatonin use is urgently required.

### 5.3. Resveratrol

Resveratrol, or trans-3,5,4′-trihydroxystilbene, is a bioactive molecule with pleiotropic bioactivities [147]. The use of resveratrol as a nutraceutical has been evaluated in many disorders in both human trials and animal models [177,178]. More importantly, resveratrol has been considered as a reprogramming strategy for preventing MetS programming [179].

Early-life resveratrol treatment has been reported to protect offspring against hypertension [64,150,151], hyperlipidemia [37,180,181], obesity [79,117,181], and insulin resistance [117,182] in various developmental programming models. Resveratrol can act as an antioxidant against oxidative stress [182]. The implication of perinatal resveratrol therapy in alleviating oxidative stress-induced features of MetS is evidenced by the protection against hypertension in a high-fructose diet model, [156] and insulin resistance in a protein restriction model [117]. Additionally, resveratrol can mediate nutrient-sensing signals as it has been considered as a SIRT1 or AMPK activator [177]. We previously reported that AMPK activation by resveratrol prevented the elevation of offspring hypertension programmed by a high-fructose diet [150] and a high-fat diet [151].

### 5.4. Probiotics/Prebiotics

In view of the fact that gut microbiota dysbiosis is an important mechanism involved in the pathogenesis of developmentally programmed MetS, it seems logical to apply probiotics or prebiotics supplementation as a potential reprogramming intervention in regards to MetS of developmental origins. In clinical practice, the most commonly used gut microbiota-targeted interventions are probiotics and prebiotics. A recent systematic review demonstrated that probiotics intake in patients with MetS improved several features of MetS, including obesity, hypertension, glucose metabolism, and dyslipidemia in some studies [183]. However, limited information is available regarding their impact on the developmental origins of MetS in humans. Using rodent models induced by a maternal high-fructose diet [39] or high-fat diet [121], supplementation with the probiotic *Lactobacillus casei* or prebiotic inulin during gestation and lactation has shown to benefit hypertension in adult offspring. Another study revealed that maternal oligofructose supplementation attenuated insulin resistance and dyslipidemia induced by maternal high-fat/-sucrose diets [141].

There are other gut microbiota-related modalities applied to prevent CVD of developmental origins, as we reviewed elsewhere [106]. As a postbiotic, acetate supplementation within gestation and lactation was reported to protect offspring against high-fructose diet-induced hypertension, a major hallmark of MetS [134]. However, its reprogramming effects on other characteristics of MetS are awaiting further clarification. Future work in other gut microbiota-target modalities is needed to better identify ideal reprogramming interventions for the developmental origins of MetS.

### 5.5. Amino Acids

Amino acids can interfere with BP regulation [156], insulin signaling, and body composition [184]. As reviewed elsewhere, several amino acid supplementations during gestation and lactation have shown to benefit hypertension of developmental origins in various rodent models [156]. These amino acids include arginine [185], taurine [186], citrulline [187], cysteine [188], and branched chain amino acids (BCAAs) [189].

As citrulline can be converted to arginine, oral citrulline supplementation has been evaluated as an add-on therapy to increase NO production [190]. Apart from preventing hypertension, citrulline supplementation has also been shown to prevent hypertriglyceridemia and attenuate liver fat accumulation programmed by a maternal high-fructose diet [191]. N-acetylcysteine, a stable cysteine analog, has shown beneficial effects for CVD [192], the major complication of MetS. Although BCAA supplementation in pregnancy is able to prevent maternal caloric restriction-induced programmed hypertension [189], another study revealed that supplementation of BCAAs in rats fed with a high-fat diet contributes to development of obesity-associated insulin resistance [193]. Considering the complexity of amino acid metabolism between the mother and the fetus in pregnancy, we must elucidate the pathophysiologic roles of specific amino acids and their interactions in the developmental programming of MetS to avoid unintended long-term consequences.

### 5.6. Others

Currently, RAS inhibitors are the first-line pharmacological therapy for hypertension, which is a major hallmark of MetS [194]. There is a shortage of data with regard to MetS of developmental origin in humans. However, animal studies revealed several RAS-based interventions that benefit in protecting against programmed hypertension [104]. Much work is needed to elucidate whether these RAS-based interventions are also effective for other characteristics of MetS.

Additionally, statins are a class of drugs recommended for dyslipidemia in MetS. In a maternal high-fat model, pravastatin administration protected offspring against obesity, hypertension [195], and dyslipidemia [196]. Further characterization of statins’ reprograming effects against all features of MetS is required in other programming models.

Furthermore, some medications targeting nutrient-sensing signals have been shown to have a positive effect with regard to MetS. A growing body of evidence indicates that PPARs may serve as therapeutic targets for treating MetS-related diseases [197]. Several PPARγ agonists have shown beneficial effects on hypertension of developmental origins [146]; however, there is still limited information to decide the appropriate PPAR ligands for reprograming all characteristics of MetS in all models. Adenosine analog 5-aminoimidazole-4-carboxamide-1-beta-D-ribofuranoside (AICAR), a direct AMPK activator, has been shown to improve glucose tolerance and lipid profiles, and to reduce BP in adult obese Zucker rats [198]. The reprogramming effect of AICAR on programmed hypertension has been observed in a maternal high-fat diet model [199]. It will be interesting to see whether AMPK activators show any benefits against other characteristics of MetS in various models of developmental programming.

## 6. Conclusions and Future Perspectives

This review highlighted the variety of adverse environmental factors during fetal development reported throughout the literature linking MetS of developmental origin, despite prior reviews that were mainly focused on nutritional imbalance [6,7,8,9].Reflecting current knowledge, our review also shed light on the fundamental mechanisms behind MetS of developmental origins at the molecular level. It opened a new window for preventing or delaying the onset of MetS via innovative reprogramming approaches.

Regardless of recent advances in establishing appropriate animal models for MetS of developmental origins, only a few models display the full manifestations of MetS. Although many reprogramming interventions have led to substantial progress in certain features of MetS in one model, attention must be paid to clarifying whether their effects are also beneficial for other MetS phenotypes; translation from one model into other models will be an additional challenge. Notably, almost no studies have taken a life course approach to determine all features of MetS in one experiment. In view of the fact that various characteristics of MetS appear throughout the course of a lifetime, most reprogramming interventions studied in a given time might be incomplete and this makes it difficult to predict their long-term effects. Meanwhile, even known reprogramming interventions appear promising, although the dose, timing and frequencies of intervention await identification and clinical validation.

## Figures and Tables

**Table 1:** Rodent models of MetS of developmental origins, categorized according to environmental factors.

Animal Models	Timing	Experimental Animal	Offspring Outcomes Relating to MetS
Nutritional imbalance			
Caloric restriction	Pregnancy and lactation	SD rats [30]/Wistar rats [31,32]	↑BP 12-16 wk [30,31], ↓insulin levels and exhibit insulin resistance 14 wk [32]
Protein restriction	Pregnancy	Wistar rats	↑BP 12 wk [33], exhibit insulin resistance 12 wk [34]
High-fat diet	Pregnancy and lactation	SD rats	↑BP 16 wk [35], ↑adiposity 16 wk [36], dyslipidemia 16 wk [37], ↑BW, exhibit dyslipidemia and hyperinsulinemia 100 day [38]
High-fructose diet	Pregnancy and lactation	SD rats/C57BL6J mice	↑BP [39], exhibit insulin resistance and dyslipidemia 12 wk [40], ↑BP, exhibit insulin resistance and obesity 1 year [41]
High-fructose diet plus high-fat diet	Pregnancy and lactation	SD rats [42]/Wistar rats [43]	↑BP 16 wk [42], ↑BW and adiposity 150 days [43]
Calcium-deficient diet	Pregnancy	WKY rats [44]/SD rats [45]	↑BP 1 year [44], ↑adiposity, exhibit insulin resistance and dyslipidemia [45]
Zinc-deficient diet	Pregnancy and lactation	Wistar rats [46]/SD rats [47]	↑BP 12 wk [46], ↑BW, exhibit insulin resistance 15 wk [47]
Vitamin D restricted diet	Pregnancy and lactation	SD rats	↑BP 8 wk [48], exhibit insulin resistance 16 wk [49]
Maternal illness/condition			
Uteroplacental insufficiency	Pregnancy	WKY rats [50]/Wistar rats [51]	↑BP 22 wk [50], exhibit dyslipidemia and insulin resistance 30 wk [51]
Polycystic ovary syndrome	Pregnancy	Wistar rat [52], SD rats [53]	↑BP 120 days [52], exhibit dyslipidemia 16 wk [53]
Maternal hypoxia	Pregnancy	Wistar rats [52]/SD rats [54]	↑BP 4 mo [52], ↑BW and adiposity, exhibit insulin resistance 12 wk [54]
Maternal inflammation	Pregnancy	SD rats [55]/Wistar rats [56]	↑BP 12 wk [55], insulin resistance 75 days [56]
Diabetes	Pregnancy	SD rats [57]/Wistar rats [58,59]	↑BP 12 wk [57], ↑BW and adiposity 12 wk [58], exhibit insulin resistance and dyslipidemia 16 wk [59]
Chronodisruption	Pregnancy and lactation	SD rats [60]/Wistar rats [61]	↑BP 12 wk [60], exhibit insulin resistance 18 wk [61]
Chemical/medication exposure			
DEHP	Pregnancy	Wistar rats	↑BP 21 wk [62], exhibit insulin resistance 80 day [63]
BPA	Pregnancy and lactation	SD rats	↑BP 16 wk [64], exhibit insulin resistance 6 mo [65]
Alcohol	Pregnancy	SD rats	↑BP 6 mo [66], exhibit insulin resistance 6 mo [67]
Nicotine	Pregnancy	Wistar rats	↑BP 8 mo [68], ↑BW and adiposity, exhibit insulin resistance [69]
Glucocorticoid	Pregnancy and postnatal days1-3	SD rats [70,71]/Wistar rats [72]	↑BP 12 mo [70,71], exhibit insulin resistance 6 mo [72]

SD = Sprague-Dawley rat; WKY = Wistar-Kyoto rat; BP = blood pressure; BW = body weight; wk = week; mo = month; di-DEHP = (2-ethylhexyl) phthalate; BPA = bisphenol A; ↑ = increased; ↓ = decreased.

## Data Availability

All data are contained within the article.
